# Trends in the Diagnosis of Gestational Diabetes Mellitus

**DOI:** 10.1155/2016/5489015

**Published:** 2016-04-12

**Authors:** Surabhi Mishra, Chythra R. Rao, Avinash Shetty

**Affiliations:** Department of Community Medicine, Kasturba Medical College, Manipal University, Manipal, Karnataka 576104, India

## Abstract

*Introduction*. Gestational diabetes mellitus (GDM) is defined as carbohydrate intolerance of variable degree with onset or recognition during pregnancy. As prevalence of diabetes is linked to impaired glucose tolerance during antenatal period, routine antenatal screening of GDM is required. However, screening tests for GDM remain controversial.* Objective*. To review different diagnostic criteria for GDM.* Materials and Methods*. Freely accessible, full-text articles from 1964 to 2015, available in PubMed in English language, pertaining to screening of GDM were reviewed.* Results*. First diagnostic criteria for GDM in 1964 by O'Sullivan and Mahan, modified by the National Diabetes Data Group (NDDG) in 1979 and Carpenter in 1982. The cut-off value as per WHO definition of GDM was 140 mg/dL, 2 hours after 75 g glucose intake. Diabetes in Pregnancy Study Group India (DIPSI), in 2006, endorsed WHO criteria but irrespective of the last meal timings. Being cost-effective, it formed the basis of national guidelines for Indians in 2014.* Conclusions*. As typical clinical scenarios are usually varied, practical guidelines that meet the constraints of low-resource settings like India are required.

## 1. Introduction

Gestational diabetes mellitus (GDM) is defined as carbohydrate intolerance of variable degree with onset or recognition during pregnancy. As per the International Diabetes Federation (IDF), Diabetes Atlas 2015, one in seven births are affected by GDM [1]. India, being the second leading dweller of diabetic subjects (69.2 million), has become the “diabetes capital of the world” harbouring around four million women with GDM alone [2]. IDF 2013 estimated a total of 21.4 million live births to be affected with hyperglycaemia in pregnancy [3]. Though the number of live births affected by hyperglycaemia in pregnancy has decreased slightly from 21.4 million to 20.9 million in 2015, the adverse perinatal outcomes in 85.1% of cases are still due to GDM, 7.4% due to other types of diabetes first diagnosed in pregnancy, and the remaining 7.5% of cases due to diabetes detected prior to pregnancy [1].

## 2. Importance of Screening for GDM

A pregnant woman diagnosed to have GDM is at an increased risk for developing Type II diabetes in the future. In addition, evidence is now emerging that women with past history of GDM also have higher prevalence of metabolic syndrome and cardiovascular diseases in comparison to those who had normal glucose tolerance during their antenatal period [4]. A recent study reported that GDM can be responsible for almost 19–30% of Type II diabetes mellitus among Saskatchewan first nation's residents in Canada [5]; another study revealed that almost half of the proportion of diabetes (47.2%) in youth can be attributed to maternal GDM in any population [6]. In the past two decades, as the age at onset of diabetes has been declining and age at marriage and child bearing is increasing, a larger fraction of women are expected to enter pregnancy with preexisting diabetes, thus creating a vicious cycle in which diabetes begets more diabetes [7, 8].

This perpetuation of the vicious cycle influencing the present prevalence of diabetes is well substantiated by the hypothesis “*foetal origin of adult disease.*” It states that glucose intolerance in continuum during pregnancy predisposes the offspring to a higher risk not only for immediate complications such as macrosomia, hypoglycemia, jaundice, respiratory distress syndrome, polycythemia, and hypocalcemia but also for long-term complications such as childhood obesity, impaired glucose tolerance in adolescence, and overt diabetes and hyperlipidemia in later life [9–11]. If the child is a girl, there is an additional risk of her developing pregestational diabetes mellitus (pre-GDM) and GDM. Lifestyle modifications or mere medical interventions late in life are of little help as they target only the postprimary prevention of overt diabetes. Thus, the need of the hour is to consider the primary preventive measures right at the commencement of intrauterine life, when the foetus begins to get exposed to various adverse intrauterine stressors like glucose intolerance, which would then continue throughout life [12].

Therefore, in view of the dramatic increase in obesity and diabetes in the present scenario, women with GDM become the ideal group for targeting primary prevention strategies for diabetes in any community. Thus, well-timed screening of all pregnant women for glucose intolerance, early diagnosis, and prompt and adequate treatment during pregnancy become worthwhile. Thereby, this concept of foetal programming highlights the importance of pregnancy which offers the platform to provide appropriate maternal health care services not only to reduce maternal and perinatal morbidity and mortality indicators, but also to prevent other intergenerational chronic diseases like diabetes, obesity, hypertension, cardiovascular diseases, and stroke [4]. Although there is enough emphasis and need for GDM screening, even today, the choice of screening criteria and the ideal timing for screening remain controversial. Therefore, the current study was designed to review the various available diagnostic criteria for GDM from past to present.

## 3. Materials and Methods

Freely accessible, full-text articles, available in PubMed and Google Scholar in English language, from 1964 to 2015 pertaining to screening for GDM were reviewed.

## 4. Results and Discussion

### 4.1. Evolution of Screening Trends

**Figure 1:**
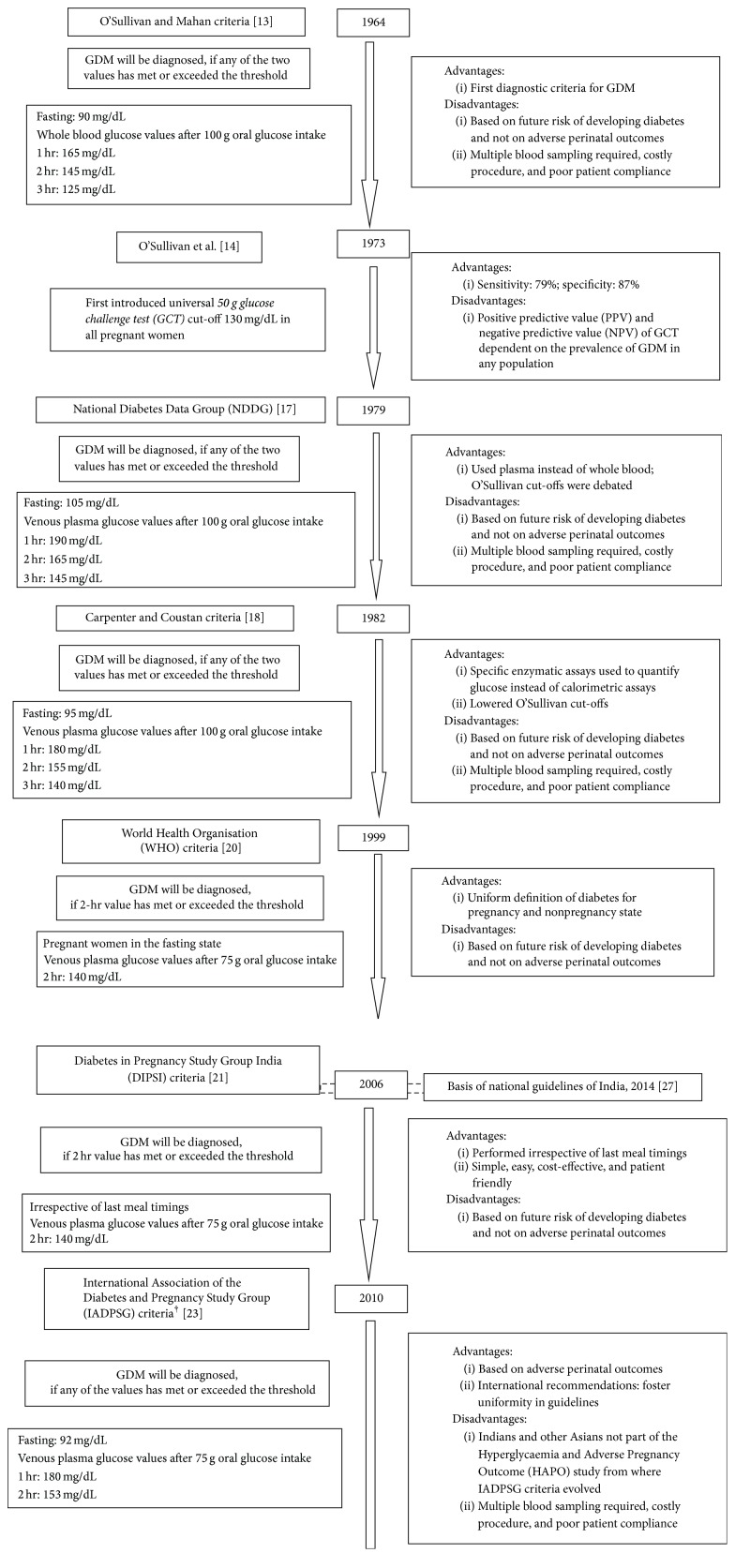
Evolution of different diagnostic criteria for GDM.

In 1964, O'Sullivan and Mahan, realizing that pregnancy had measurable effects on the metabolism of carbohydrates, gave guidelines for the diagnostic criteria of GDM based on their results performed on 752 pregnant women by 3-hour 100 g oral glucose tolerance test (OGTT) [13]. If any two threshold values for whole blood glucose after a 100 g OGTT using Somogyi-Nelson method were met or exceeded, gestational diabetes was diagnosed as shown in Figure 1. The main drawback of O'Sullivan and Mahan criteria was that the glycaemic cut-offs used for diagnosing GDM were originally validated against the future risk of these women developing diabetes and not on foetal outcomes. In 1973, O'Sullivan et al. suggested the use of a 50 g 1-hour oral glucose challenge test (GCT) to screen for gestational diabetes [14]. Using venous whole blood samples and Somogyi-Nelson method, they found that a threshold of 130 mg/dL was 79% sensitive and 87% specific for gestational diabetes in a population of 752 pregnant women, all of whom also underwent the diagnostic 100 g 3-hour OGTT. While the positive predictive value (PPV) was 14%, the negative predictive value (NPV) was 99.4%. This meant that 0.6% of individuals with normal screening tests had gestational diabetes. These two attributes of a screening test, unlike sensitivity and specificity, are highly dependent on the prevalence of the GDM in any population. If the prevalence of GDM had been 10% instead of 2.5% as in O'Sullivan's population, the NPV would have decreased to 97.5%, meaning that 2.5% of individuals with 1-hour glucose values would still have GDM with threshold of 130 mg/dL. Since the study population underwent both the screening and diagnostic test, O'Sullivan study provided complete ascertainment of the results [15]. However, in 1986, the American Congress of Obstetricians and Gynecologists (ACOG) recommended GCT screening only for women with risk factors like age above 25 years, race/ethnicity of high diabetes prevalence, or family history of Type II diabetes in first-degree relatives [16]. But screening for conventional risk factors of GDM by means of history taking was relatively insufficient as it was found that a significant number of the GDM women do not possess these risk factors.

Soon, the National Diabetes Data Group (NDDG) reported that the glycaemic cut-off values proposed by O'Sullivan and Mahan were derived when measured in whole blood using Somogyi-Nelson method. Thus, in 1979, NDDG preferred the use of plasma instead of whole blood for glucose analysis [17]. As plasma glucose concentration is 11% higher than in whole blood (owing to higher concentration of water in plasma), NDDG raised the glycaemic cut-offs and proposed new guidelines for diagnosing GDM. If any two or more plasma glucose values after 100 g OGTT were met or exceeded the upper limits, gestational diabetes was diagnosed, as depicted in Figure 1 [17].

After three years, in 1982, Carpenter and Coustan observed that Somogyi-Nelson method used calorimetric assays to quantify glucose as done by O'Sullivan and Mahan. But recognizing the limitation of the method, Carpenter and Coustan replaced the calorimetric assays by more specific enzyme assays. This enabled them to further lower the NDDG cut-offs and GDM was diagnosed if any two or more values met or exceeded the cut-offs (Figure 1) [18].

Till then, NDDG used the term “diabetes or impaired glucose intolerance” to define GDM instead of “carbohydrate intolerance of variable severity” except when in 1985 at the Second International Workshop-Conference on Gestational Diabetes Mellitus, where the new terminology was accepted to define GDM [17, 19]. But the World Health Organisation (WHO) still favoured the use of the former terminology with the underlying concept that there should be a uniform definition of diabetes in all populations. Thus, in 1999, WHO recommended the use of cut-off values which define glucose intolerance and diabetes with or without pregnancy. The diagnosis of GDM was made on a single cut-off value of 140 mg/dL (7.8 mmol/L) when 2-hour 75 g postglucose load was measured after overnight fasting (>200 mg/dL: overt diabetes during pregnancy) (Figure 1) [20].

But it was soon realised that this criterion requires antenatal women to be in the fasting state. But most of the time pregnant women do not come fasting because of the probable belief that they should not fast for long hours which makes this criterion highly impractical in clinical settings. Moreover, the drop-out rate becomes higher if a woman is asked to come again the next day for OGTT. Thus, in 2006, Diabetes in Pregnancy Study Group India (DIPSI) used the modified version of WHO in which the same 75 g 2 hr OGTT venous plasma glucose was measured in a nonfasting state at 24–28 weeks of gestation irrespective of the last meal timings. It was found to be as efficacious as the test done in fasting state as per recommendations of the WHO (Figure 1) [21].

### 4.2. Recent Trends in Screening

Although it is nearly 40 years since the inception of O'Sullivan and Mahan's criteria for the diagnosis of GDM, still there is no uniform consensus for the same. The major reason is that the criteria were designed to identify pregnant women who are at high risk for developing subsequent diabetes after pregnancy rather than those who are at increased risk for adverse perinatal outcomes. There is consensus that overt diabetes during pregnancy, whether symptomatic or asymptomatic, is associated with significantly increased risks of adverse pregnancy outcomes. Thus, in 2008, Hyperglycaemia and Adverse Pregnancy Outcome (HAPO) study was conducted which proved that the risks of adverse pregnancy outcomes are significantly associated with varying degrees of maternal hyperglycaemia that are less severe than that in overt diabetes mellitus [22]. Soon, in 2010, International Association of the Diabetes and Pregnancy Study Group (IADPSG) considered and reviewed the study results of HAPO study and released new international guidelines for diagnosing GDM with an idea to foster uniform intercontinental approach in the field of diabetes in pregnancy (Figure 1) [23]. But it was later realised that HAPO study was performed mainly among Caucasian population; many Asian countries including India were not part of the HAPO study.

Though IADPSG guidelines were undervalued especially in Indian settings, a prospective, collaborative study performed in 2012 among a cohort of 1,463 consecutive pregnant women at a community health centre in Chennai proved that there exists no statistical significant difference between the current guidelines for diagnosing GDM recommended by DIPSI or IADPSG (*p* = 0.21, by McNemar Test) such that even IADPSG can be adopted for subjects of Indian origin [24]. The difference in the diagnostic capability between the two criteria in diagnosing GDM was proved to be 1.2% (*p* > 0.02). On similar grounds, Chennai study findings also indicated that OGTT is not necessary to diagnose a case of GDM [24]. Not only are multiple blood samples inconvenient and costly for the population, but also they reduce the patient compliance and thus reduce the rate of GDM. Instead, single blood draw at 2 hours following 75 g OGTT not only is a patient friendly procedure but also reduces the cost of analysis especially in high-risk population where GDM can manifest itself in all trimesters of pregnancy [25, 26]. Moreover, on using DIPSI procedure, 28% of the pregnant women who were missed in the first visits because of their normal OGTT were diagnosed to have GDM on subsequent visits [25]. Thus, DIPSI procedure also proved reliable in diagnosing GDM besides being patient friendly and cost-effective as compared to the more stringent IADPSG criteria.

**Figure 2:**
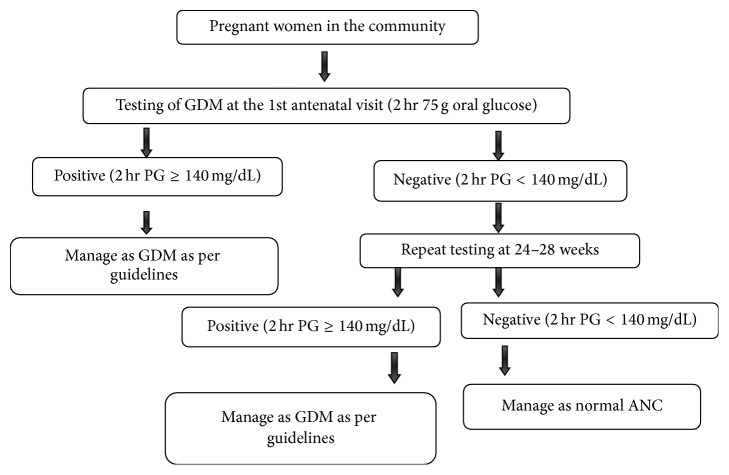
National Guidelines for Diagnosis and Management of Gestational Diabetes Mellitus, Government of India, 2014 [27].

Hence, in 2014, keeping in view the differences in national and international guidelines for diagnosing GDM, the Government of India (GOI) supported DIPSI guidelines and recently formulated their own national guidelines based on DIPSI diagnostic criteria (Figures 1 and 2) [27]. They recommended universal screening of all Indian pregnant women twice during their entire antenatal period by following one-step procedure using 2-hour 75 gm OGTT irrespective of their last meal timings. The purpose of implementing universal screening in a country like India is to rule out the likelihood of pregnant women suffering from undiagnosed Type II diabetes/pre-GDM when they walk in for the first time to an antenatal clinic unaware of their prepregnancy glycaemic levels [27]. This situation practically holds true in a place like India which ranks highest among South-Asian countries with respect to the prevalence of GDM [28] and where awareness among rural Indian women is expected to be suboptimal [29]. The national guidelines for the diagnosis of GDM recommend 75 g glucose to be given after dissolving in approximately 300 mL water irrespective of the last meal timings. The intake of the solution should be completed within five minutes. In case vomiting occurs within 30 minutes of oral glucose intake, the test has to be repeated the next day. But if it occurs after 30 minutes, the test continues. The first test is to be done early in pregnancy and the second between 24 and 28 weeks of gestation. There should be a minimum gap of four weeks between the two tests. If the woman presents beyond 28 weeks of gestation, only one test is to be done at the first contact. The national guidelines also mandate the availability of plasma calibrated glucometer at all antenatal clinics located at medical colleges, district hospitals, and other Comprehensive Emergency Obstetric Care Services (CEmOC) centres in order to evaluate blood glucose 2 hours after the oral glucose load instead of semiautoanalyser or autoanalyser. This is to ensure quick interpretation of results which can be made available to the subject on the same day [27].

### 4.3. Current Practice in Public Health Setup

However, the practice of the abovementioned guidelines in primary care facilities of India is still unclear. In Tamil Nadu, a recent community-based study revealed prevalence of GDM 17.8% in urban, 13.8% in semiurban, and 9.9% in rural areas but, in public health facilities at Bangalore, it was found to be <1% [30, 31]. This underestimation of GDM prevalence in public health facilities was explained to an extent by the facts revealed in District Level Health Survey (DLHS). As per DLHS-4 (2012-13), only 59% of the government health services were utilised in Bangalore (46% in Udupi) for providing complete antenatal check-up to a pregnant woman [32]. Only 70% of the public health facilities available in Bangalore provided laboratory facilities to screen GDM, although the choice of screening test varied among them. Only 46% of the public health doctors employed OGTT for screening GDM. But even among them, the amount of glucose used for OGTT varied. In addition, the recommended time interval of glucose testing at 1, 2, and 3 hrs was adhered to only by 58%. Mere one-tenth of the doctors (8%) preferred 100 gms glucose while around three-fourths (74%) prescribed 75 gms for OGTT and the remaining 18% employed 50 gms for OGTT [31].

## 5. Conclusions

It has been found that most of the screening protocols are often derived from the evidence of scientific research that are carried out by academic institutions which are not only well-equipped but also well-resourced. However, the typical clinical settings especially public health care facilities of low- and middle-income countries face conditions that are often far away from these ideal settings. Thus, the choice of diagnostic criteria should be the most appropriate and cost-effective and be able to cover the major section of the population. Irrespective of the clinical settings, whether urban or rural, public or private, the diagnostic criteria should be able to meet the needs and constraints of low-resource settings especially India, where there is pressing demand for clear and practical guidelines.
